# Biomimetic Nanoparticles Potentiate the Anti-Inflammatory Properties of Dexamethasone and Reduce the Cytokine Storm Syndrome: An Additional Weapon against COVID-19?

**DOI:** 10.3390/nano10112301

**Published:** 2020-11-20

**Authors:** Roberto Molinaro, Anna Pasto, Francesca Taraballi, Federica Giordano, Jamil A. Azzi, Ennio Tasciotti, Claudia Corbo

**Affiliations:** 1 Center for Biomimetic Medicine, Houston Methodist Research Institute, Houston, TX 77030, USA; molinaro.roberto@hsr.it (R.M.); anna.pasto@humanitasresearch.it (A.P.); tasciottiennio@gmail.com (E.T.); 2 IRCCS San Raffaele Hospital, 20132 Milan, Italy; 3 Center for Musculoskeletal Regeneration, Houston Methodist Academic Institute, Houston, TX 77030, USA; ftaraballi2@houstonmethodist.org (F.T.); fgiordano@houstonmethodist.org (F.G.); 4 Orthopedics and Sport Medicine, Houston Methodist Hospital, Houston, TX 77030, USA; 5 Renal Division, Brigham and Women’s Hospital, Harvard Medical School, Boston, MA 02115, USA; jazzi@bwh.harvard.edu; 6 Biotechnology Program, San Raffaele University and IRCCS San Raffaele Pisana, 00166 Roma RM, Italy; 7 Department of Medicine and Surgery, Center for Nanomedicine Nanomib, University of Milan Bicocca, 20854 Vedano al Lambro MB, Italy

**Keywords:** COVID-19, inflammation, leukocyte-like carriers, cytokine storm, biomimetic nanovesicles

## Abstract

Recent studies on coronavirus infectious disease 2019 (COVID-19) pathophysiology indicated the cytokine release syndrome induced by the virus as the main cause of mortality. Patients with severe COVID-19 infection present a systemic hyper inflammation that can lead to lung and multi-organ injuries. Among the most recent treatments, corticosteroids have been identified to be effective in mitigating these catastrophic effects. Our group has recently developed leukocyte-derived nanovesicles, termed leukosomes, able to target in vivo the inflamed vasculature associated with pathological conditions including cancer, cardiovascular diseases, and sepsis. Herein, to gain insights on the anti-inflammatory properties of leukosomes, we investigated their ability to reduce uncontrolled inflammation in a lethal model of lipopolysaccharide (LPS)-induced endotoxemia, recapitulating the cytokine storm syndrome observed in COVID-19 infection after encapsulating dexamethasone. Treated animals showed a significant survival advantage and an improved immune response resolution, as demonstrated by a cytokine array analysis of pro- and anti-inflammatory cytokines, chemokines, and other immune-relevant markers. Our results showed that leukosomes enhance the therapeutic activity of dexamethasone and better control the inflammatory response compared to the free drug. Such an approach could be useful for the development of personalized therapies in the treatment of hyperinflammation related to infectious diseases, including the ones caused by COVID-19.

## 1. Introduction

In December 2019, the virus causing coronavirus disease, i.e., COVID-19, was first identified in Wuhan, China. Even though Wuhan was locked down in January 2020, we have experienced a rapid, uncontrolled global spread of the virus, causing a pandemic. When not asymptomatic, patients affected by COVID-19 can present symptoms of hyper inflammation associated with a cytokine storm syndrome, ultimately leading to respiratory dysfunction syndrome and death. Pathophysiological features are dominated by an acute pneumonic process with alveolar damage and inflammatory infiltrates [1]. Indeed, a subgroup of patients show high levels of inflammatory markers, e.g., C-reactive protein, IL-1, and IL-6. As a matter of fact, several therapeutic options are based on the mitigation of inflammatory organ injury in viral pneumonia. Despite no specific drugs/antivirals having been identified, physicians are experimenting with the anti-inflammatory effects of corticosteroids in an attempt to prevent and/or treat these catastrophic effects. In this context, randomized multicenter trials in COVID-19 patients have revealed that patients treated with dexamethasone showed a lower mortality rate compared to those who received standard treatment [1,2].

The inhibition of the body’s inflammatory responses to pathological conditions is challenging and the development of innovative approaches for an effective treatment is crucial. In this context, drug delivery systems, such as nanoparticles (NPs), that improve the delivery of drugs to the target sites, represent a valid therapeutic option that have attracted increasing attention.

The recent concept of bio-inspired nanocarriers [3,4,5,6,7] led to the introduction of a variety of cell membrane-coated nanoparticles for several applications including vaccination [8], detoxification [9], and targeted drug delivery [10,11,12]. In this context, macrophage-like NPs, synthesized by wrapping a poly(lactic-co-glycolic acid) (PLGA) core with cell membrane derived from macrophages, have been recently used by Zhang’s group as a sort of “sponge” for mechanical endotoxin removal as a potential tool for the management of inflammatory response due to sepsis [13]. In their work, the authors showed that these macrophage-like NPs sequester pro-inflammatory cytokines and inhibit their ability to potentiate the sepsis cascade [13]. Similarly, iron oxide nanoclusters wrapped with macrophage membranes were tested in an in vivo model of endotoxemia, a severe pathophysiology induced by lipopolysaccharide LPS, which causes high mortality rates in clinic due to life-threatening syndromes, such as sepsis [14]. Our group has recently developed leukocyte-inspired nanovesicles, called leukosomes, which, by mimicking real cells, avoid rapid clearance by the mononuclear phagocytic system and target the inflamed endothelium [11,15,16]. Due to these properties, we tested them for the treatment of inflammatory-based diseases, such as cardiovascular diseases [17] and cancer [5,18,19]. We demonstrated that these nanovesicles exhibited an intrinsic anti-inflammatory activity in mouse models of lipopolysaccharide (LPS)-induced localized [11] and systemic [20] inflammation and inflamed bowel disease [21]. Indeed, compared to the other biomimetic approaches described above, where LPS was mechanically removed from the circulation, we demonstrated both in vitro and in vivo that the anti-inflammatory features of leukosomes are due to a direct interaction with macrophages. This interaction leads to an increased release of anti-inflammatory cytokines, while reducing the pro-inflammatory ones, thus decreasing the systemic inflammatory response and prolonging mouse survival. Moreover, it is worth noting that these anti-inflammatory properties derive from the empty carriers, without the need of a payload, which behave like anti-inflammatory exosomes, with whom they share similar size and surface charge, and comparable protein profiles, but a more potent anti-inflammatory activity [20].

Based on these results, and considering the impact of dexamethasone in COVID-19 therapy, we tested the anti-inflammatory properties of leukosomes loaded with dexamethasone in an LPS-induced mouse model of endotoxemia that recapitulates the cytokine storm syndrome typical of some systemic infections, including the catastrophic recent one causing the COVID-19 pandemic.

Our results showed that, when compared to dexamethasone alone (free DEX), dexamethasone-loaded leukosomes (DEX-LKs) modulated the plasma concentration of immune-relevant markers (e.g., cytokines, chemokines and complement factors) and enabled prolonged survival. This is mainly due to the ability of DEX-LKs to reduce the expression of typical pro-inflammatory markers, such as TNF-α, IL-16 and IL-1α, and to modulate the expressions of those chemokines involved in the recruitment of T and B cells to the pulmonary tissue, thus exacerbating the local inflammatory response.

## 2. Materials and Methods

### 2.1. Synthesis and Characterization of Nanoparticles

Leukosomes were synthesized according to the protocols designed in our laboratory and previously described [22]. Briefly, 1,2-dipalmitoyl-sn-glycero-3-phosphocholine (DPPC), 1,2-dioleoyl-sn-glycero-3-phosphocholine (DOPC) and cholesterol (4:3:3 molar ratio) were mixed with J774 macrophage membranes using the NanoAssemblr™ platform (Precision Nanosystem, Vancouver, BC, Canada). Cyclodextrin-complexed dexamethasone (DEX) was added to the aqueous phase during assembly. Unencapsulated free DEX was separated from the encapsulated one through ultracentrifugation at 130,000× *g* for 1 h at 20 °C, the supernatant was removed, and the pellet resuspended in PBS pH 7.4 for further experiments. The sizes and surface charges of the nanovesicles were characterized as previously reported [18]. Dynamic light scattering (DLS) analysis using a Nanosizer ZS (Malvern Instruments, Malvern, UK) was carried out to measure diameter and size homogeneity, respectively. Surface charge (Zeta potential) was measured using a ZetaSizer Nano ZS (Malvern Instruments, Malvern, UK). All the results are the average of at least 5 measurements, with 10 runs each.

### 2.2. Cells

Balb/c mouse pulmonary vein endothelial cells were cultivated in gelatin-coated plates in M1168 medium supplemented with 0.1% VEGF, 0.1% ECGS, 0.1% Heparin, 0.1% Hydrocortisone, 1% l-glutamine, 1% Antibiotics and 10% FBS (according to Cell Biologics). Mouse macrophage J774 cells were maintained in DMEM supplemented with 1% Pen/Step, 1% l-Glutamine and 10% FCS. For the in vitro assay, cells were inflamed with LPS (100 ng/mL). After 24 h, the medium was replaced, and the cells were treated with free Dexamethasone (DEX) or DEX-loaded leukosomes (both final concentration of 100 uM). Cells were collected after 24 h for qRT-PCR analysis.

### 2.3. RNA Extraction and qRT-PCR Analysis

Total RNA was extracted using RNeasy Mini Kit (Qiagen, Hilden, Germany). RNA concentration and purity were measured using the NanoDrop ND1000 spectrophotometer (NanoDrop Technologies, as described elsewhere) [20]. According to the datasheet, the cDNA was synthesized from 0.5–1 μg of total RNA (iScript retrotranscription kit, Bio-Rad Laboratories, Hercules, CA, USA). Gene expression analysis was performed using Taqman probes and the fast advanced master mix (ThermoFisher Scientific, Waltham, MA, USA) on a StepOne Plus real-time PCR system (Applied Biosystems, Foster City, CA, USA). Each sample was run in triplicate. Results were analyzed using the comparative ΔΔCt method normalized to the housekeeping β-actin and reported as mean ± SEM.

### 2.4. In Vivo Study

BALB/c mice (6–8 weeks old) from Charles River Laboratory were randomly assigned to a healthy control group (CTRL, n = 8), an untreated group (Untreated, n = 8), a group treated with dexamethasone (free DEX, n = 8), and a group treated with dexamethasone-loaded leukosomes, (DEX-LK, n = 8). Systemic inflammation was induced via an intraperitoneal injection of LPS (15 mg/Kg) in all the above groups except for the CTRL. After 30 min, DEX-loaded leukosomes or free DEX (5 mg/Kg of DEX) were systemically injected via intravenous injection (administered to the tail vein). Mice were monitored according to animal protocol guidelines for the duration of the experiments. Animal studies were carried out according to the guidelines established by the Animal Welfare Act and the Guide for the Care and Use of Laboratory Animals. The protocol was approved by Houston Methodist Research Institute’s (HMRI) Institutional Animal Care and Use Committee (AUP-0618-0037). All our efforts were devoted to reducing the number of animals used and minimizing their suffering. Blood samples were collected in tubes containing heparin for further analysis at 20 min, 2 and 8 h from treatment administration.

### 2.5. Cytokine Protein Array

A Proteome Profiler Mouse Cytokine Array Kit, Panel A (R&D system, Minneapolis, MN, USA) was employed for the profiling of chemokines, cytokines, and acute phase proteins following the LPS-induced inflammation and subsequent treatment with free DEX and DEX-LKs. This array allows for the detection of multiple molecules at the same time. The results were obtained using the manufacturer’s instructions. The protein array images were scanned and pixel density analyzed by Molecular Imager ChemiDoc XRS System + Image Lab Software v.4.1 (Bio-Rad, Hercules, CA, USA). The basal levels of plasma cytokines of non-treated mice were evaluated by the same array and used to normalize the data.

## 3. Results

### 3.1. Preparation and Characterization of Nanoparticles

**Figure 1:**
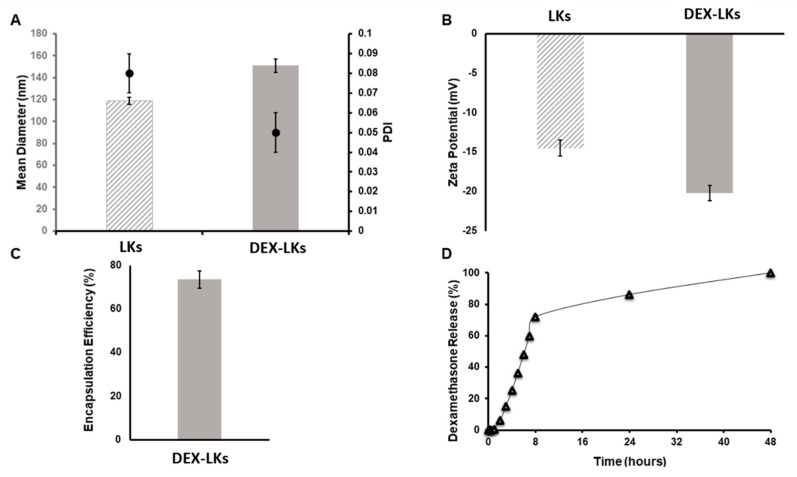
Physical and pharmaceutical characterization of leukosomes and dexamethasone-loaded leukosomes. (**A**) Dynamic light scattering analysis and polydispersity index (PDI) and (**B**) Zeta potential measurements comparing unloaded leukosomes and dexamethasone-loaded leukosomes. Data are presented as mean ± standard deviation. (**C**) Encapsulation efficiency and (**D**) release profile of DEX-Leuko in PBS at 37 °C.

Leukosomes were assembled using manufacturing protocols developed by our group that allow for a standardized, reproducible, and scalable manufacturing of biomimetic nanoparticles [22]. DLS analysis revealed that leukosomes have an average size of 120 nm (Figure 1A), a narrow distribution as showed by the polydispersity index (PDI) values ≤ 0.1 (Figure 1A), and a negative surface charge of −14 mV (Figure 1B) [11]. Dexamethasone encapsulation produced an increase in the mean diameter (average size = 150 nm) but did not significantly affect size homogeneity and zeta potential values (PDI = 0.05; ZP = −20 mV—Figure 1). The concentration of DEX into leukosomes was 1.53 µM, representing 75% of the initial amount (Figure 1C). Kinetic studies showed an initial phase characterized by a burst release of DEX, which reached around 75% at 10 h after administration, followed by a second phase where the remaining 25% was slowly released until 48 h (Figure 1D).

### 3.2. Free and Leukosome-Loaded Dexamethasone Reduces the Expression of Anti-Inflammatory Genes Both in Endothelial and Macrophage Cell Lines

Our previous data revealed that empty leukosomes are able to actively engage macrophages and induce an anti-inflammatory phenotype, thus reducing the expression of pro-inflammatory genes while increasing that of the anti-inflammatory ones [20]. Here, we asked whether leukosomes could potentiate the anti-inflammatory properties of DEX in an in vitro setting.

**Figure 2:**
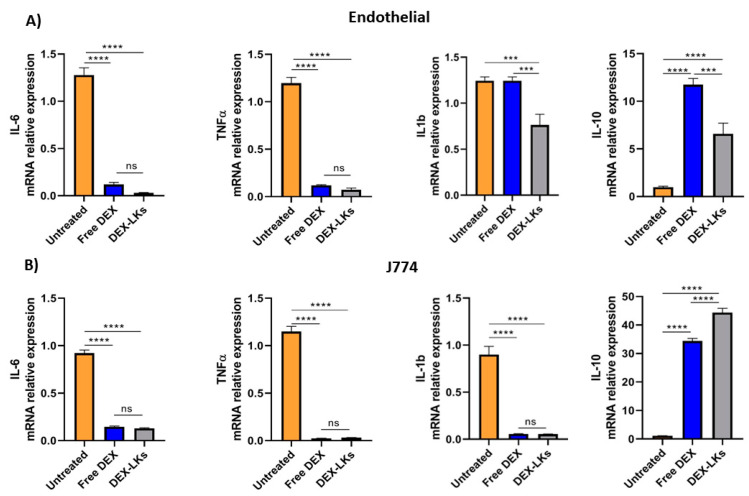
In vitro experiments. mRNA expression levels of pro and anti-inflammatory cytokines evaluated in murine endothelial (**A**) and macrophage (**B**) cells inflamed with LPS and then treated with free Dexamethasone (Free DEX), Dexamethasone-loaded leukosomes (DEX-LKs) or left untreated (Untreated). Data are expressed as mean ± SEM from three independent experiments. Asterisks depict significant differences (**** < 0.0001 and *** 0.0002). ns: not significant.

Among the hallmarks of sepsis-like systemic inflammation, endothelial cells and leukocytes have been reported to have a pivotal role [23], as suggested by the immunosuppressive state and the increased vascular permeability observed in these patients. Therefore, we focused our in vitro experiments on the effect of either free dexamethasone or dexamethasone-loaded leukosomes towards endothelial and macrophage cell lines. Our results showed that the inflammatory response following treatment with LPS was inhibited by DEX treatment in both cell lines. Indeed, compared to the untreated control, the incubation of LPS-stimulated endothelial cells (Figure 2A) and macrophages (Figure 2B) with either free DEX or DEX-LKs was associated with a decrease in the mRNA relative expression of the pro-inflammatory cytokines IL-6 and TNF-α. Interestingly, in the LPS-activated endothelial cells, treatment with DEX-LKs was associated with a higher reduction in the expression of IL-1b, compared to free DEX (Figure 2A). In addition, in both inflamed macrophages and endothelial cells, DEX-LKs significantly increased the relative expression of the anti-inflammatory cytokine IL-10 compared to untreated control (Figure 2A,B). Moreover, in J774 cells, the IL-10 induction of DEX-LKs was higher than free DEX, suggesting that in macrophages, the encapsulation in leukosomes ameliorated the anti-inflammatory effect of the drug.

Overall, these results showed that corticosteroid treatment, both as a free drug and as encapsulated within leukosomes, can reduce in vitro the expression of pro-inflammatory markers. On the other hand, the encapsulation in leukosomes greatly ameliorated the effects on the levels of the anti-inflammatory marker, IL-10.

### 3.3. Leukosomes Potentiate Dexamethasone Activity and Prolong Mouse Survival in an LPS-Induced Endotoxemia Model

**Figure 3:**
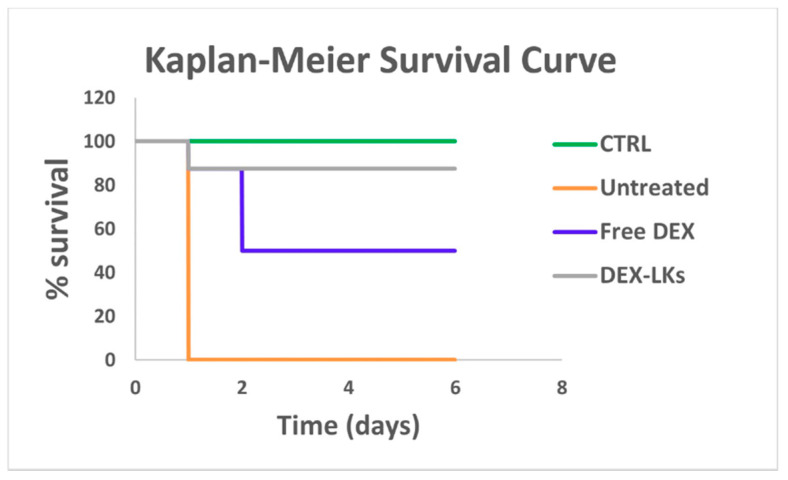
Leukosomes prolong the in vivo survival in an LPS-induced endotoxemia murine model. Kaplan–Meier survival curves of mice (n = 8) injected with LPS and treated with free dexamethasone (Free DEX) and dexamethasone-loaded leukosomes (DEX-LKs) or left untreated. Healthy mice were used as control (CTRL).

We then proceeded to evaluate the in vivo ability of DEX-LKs treatment to promote the resolution of inflammation by testing them in a mouse model of LPS-induced endotoxemia, which resembles the cytokine storm scenario characterized by elevated levels of inflammatory cytokines typical of COVID-19 infection. Notably, Kaplan–Meier survival curves revealed that treatment with DEX-LKs significantly improved mouse survival rates when compared to free DEX treatment. Indeed, 90% of DEX-LKs treated mice survived to more than 5 days, while over that time period, less than 50% of free DEX treated mice survived (Figure 3).

To demonstrate that the prolonged survival rate of DEX-LKs treated mice correlates to a resolution of an uncontrolled inflammatory response, we used an antibody array system analyzing pooled serum samples of LPS-injected mice, untreated mice, and mice that had been treated with free or leukosome-encapsulated DEX. The blood was collected i.e., 20 min, 2 h and 8 h from treatment administration. This allowed us to monitor the evolution of cytokine response (Supplementary Figure S1). In particular, we checked the variations of the plasmatic levels of the cytokines reported in Table 1 and performed a semiquantitative analysis of these detected cytokines by measuring the average levels of spot densities. We observed DEX-LKs having an effect at various levels of the immune response pathway, including modulation of the plasma levels of pro-inflammatory cytokines (ICAM-1, IL-1α, IL-16, TNFα and M-CSF) and chemokines (CXCL13, RANTES and CCL2), as well as the activity of the complement effectors (such as C5-C5a). Table 1 reports a summary of the findings for the most relevant markers identified through the protein array that were reduced in the group treated with DEX-LKs compared to the untreated and DEX-treated groups. It is worth noting that in most of the cases, DEX-LKs exerted a significant effect at 20 min after treatment administration, mainly regarding the cytokines group. On the other hand, we observed that a reduction in chemokines occurred later, between 2 and 8 h after treatments had been administered.

**Figure 4:**
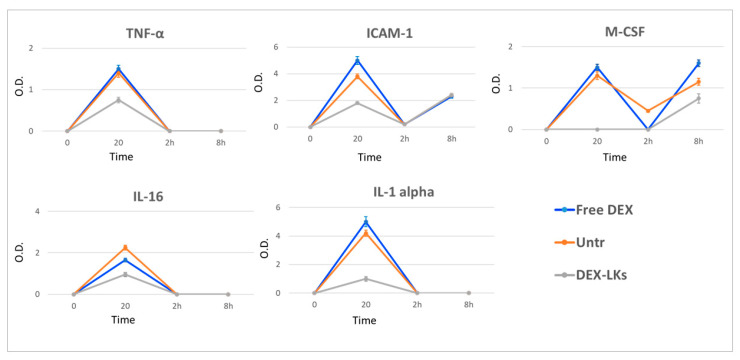
Levels of inflammatory cytokines following treatment. The average net optical intensities (O.D.) for each cytokine are shown for each group at different time points. The basal levels of plasma cytokines of non-treated mice were evaluated by the same array and used to normalize the data. Results are presented as mean ± SD (n = 4).

Several studies report that elevated plasma levels of cytokines are associated with poor outcomes in COVID-19 patients [24]. We observed that, compared to free DEX, DEX-LKs significantly reduced the plasma levels of TNFα, IL-1α, IL-16, circulating ICAM-1 and M-CSF (Figure 4).

**Figure 5:**
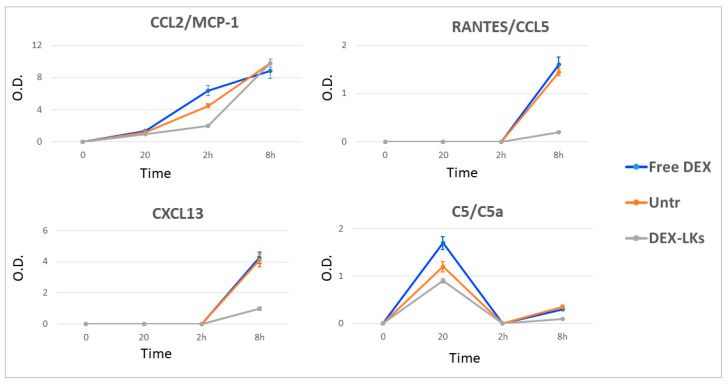
Levels of chemokines and complement proteins following treatment. The average net optical intensities (O.D.) for each chemokine are shown for each group at different time points. The basal levels of plasma cytokines of non-treated mice were evaluated by the same array and used to normalize the data. Results are presented as mean ± SD (n = 4).

Chemokines are low molecular weight proteins characterized by their powerful chemoattractant activity, which is fundamental for the immune cell recruitment typical of inflammation. Compared to the current knowledge on the cytokine storm, the role of chemokines’ dysregulation and their relationship with COVID-19 is still debated [25]. Our results revealed that DEX-LKs treatment reduced the expression of CCL2, CXCL13 and RANTES compared to free DEX treatment (Figure 5). It is worth noting that these chemokines are mainly involved in pulmonary inflammation in COVID-19 patients [25]. They have been associated with an increased inflammatory response at the level of airway epithelial cells and macrophages, and with chemotactic activity towards B and T cells [26]. Lastly, we observed a reduction in the terminal complement effector C5 in the group treated with DEX-LKs (Figure 5). Complement activation exerts a fundamental role in the immunopathology of COVID-19. In particular, the complement factor C5 and its receptor, known as CD88, play a fundamental role in the initiation and maintenance of inflammatory responses due to their involvement in the recruitment and activation of neutrophils and monocytes. As a matter of fact, the levels of C5a are proportional to the increased severity of COVID-19. High levels of CD88 were found in blood and pulmonary myeloid cells, thus highlighting their role in the pathophysiology of acute respiratory distress syndrome. In fact, it has been recently demonstrated that an inhibition of this axis can limit the infiltration of myeloid cells into damaged organs and prevent lung inflammation in COVID-19 patients with acute respiratory distress syndrome [27].

Finally, our study showed that neither treatment had any effect on CXCL2 and IL-6 compared to the untreated control (Supplementary Figure S2). In particular, IL-6 plasma levels were higher at both 2 and 8 h after treatment administration for mice treated with DEX-LKs compared to the free DEX group, thus revealing that under these specific experimental conditions survival rates were independent of the levels of IL-6.

Taken together, in agreement with the most recent studies on this topic and the observations reported by the physicians, our results demonstrated that corticosteroid treatment favors the acceleration of the immune response activation resolution. Notably, we demonstrated that the encapsulation of dexamethasone in biomimetic nanoparticles can improve its anti-inflammatory effect, attenuating the cytokine storm.

## 4. Conclusions

Many pathologies originate from an uncontrolled inflammatory condition. Among these, where COVID-19 infections are initially characterized by a status of hyper inflammation accompanied by a cytokine storm, later acute respiratory distress syndrome and death can occur. Importantly, the cytokine storm has been identified as a main factor leading to a severe clinical prognosis. This concept emerged following the observation that COVID-19 patients requiring intensive care treatment had higher levels of pro-inflammatory cytokines compared to those individuals presenting a less severe form of the infection. Herein, we reported the development of biomimetic NPs encapsulated with dexamethasone for the mitigation of the hyperinflammation induced by LPS injection. We used an in vivo murine model of endotoxemia and we revealed that leukosomes loaded with dexamethasone, when compared to the free drug, showed more promising results in the resolution of immune response and in the survival rate.

In relation to COVID-19 infection, we speculate that leukosome approach to attenuating the cytokine storm syndrome may be relevant from both a pharmaceutical/therapeutic and logistic standpoint. In the first case, the leukosome approach allows us to potentiate the pharmacological efficacy of a corticosteroid drug, dexamethasone, thus preventing the escalation of events that cause patients to require intensive pharmacological treatment and lung-ventilation. On the other hand, the need for intensive care unit (ICU) treatment brought to light one of the major issues of the COVID-19 pandemic: ICU capacity and its overload risk. If a hospital runs out of intensive care unit spots, more patients will die due to being unable to access necessary treatments. Our approach could prevent the need for intensive care treatment, thus keeping ICU loads well below their maximum capacity.

## Figures and Tables

**Table 1:** List of the cytokines and chemokines detected in the array, reporting their action and their levels following treatment with DEX-LKs compared to free DEX.

Name/Abbreviation	Class	Produced by	Action	Following Treatment with DEX-LKs	
Interleukin 1 alpha/IL-1α	Cytokine	Macrophages and lymphocytes	Pro-inflammatory, promotes activation and secretion of cytokines and other acute-phase proteins	20 min	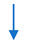
Interleukin 6/IL-6	Cytokine	Macrophages, lymphocytes, fibroblast, and others	Pro-inflammatory properties	2 h and 8 h	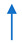
Interleukin 16/IL-16	Cytokine	T-cells, eosinophils, and mast cells	Chemoattractant, modulator of T-cell activation	20 min	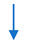
Tumor necrosis factor alpha/TNFα	Cytokine	Macrophages and lymphocytes	Differentiation and activation of cells of the immune system	20 min	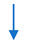
circulating adhesion molecule-1/cICAM-1	Adhesion molecule	Endothelial cells	Involved in the interaction of circulating neutrophils with vascular endothelium during inflammation.	20 min	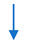
B lymphocyte chemoattractant/BLC (CXCL13)	Chemokine	B cell follicles of secondary lymphoid organs	Attracts B and T-cells to sites of infection and inflammation	8 h	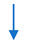
RANTES (CCL5)	Chemokine	Circulating T-cells	Pro-inflammatory	8 h	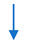
Macrophage inflammatory protein 2-alpha/MIP-2 (CXCL2)	Chemokine	Macrophages and monocytes	Chemotactic for human polymorphonuclear leukocytes	20 min	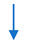
Complement component 5/C5-C5a	Complement protein	Macrophages, hepatocytes	C5a is a protein fragment highly inflammatory	20 min	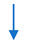
Macrophage colony-stimulating factor/M-CSF	Cytokine	Monocytes, fibroblast, others	Induces the differentiation of hematopoietic stem cells into macrophages	20 min and 8 h	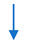
Monocyte chemotactic protein 1/(MCP1) CCL2	Chemokine	Monocytes, macrophages, and dendritic cells	Recruits monocytes, T-cells, and dendritic cells to the sites of inflammation	2 h	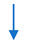

## Supplementary Materials

The following are available online at https://www.mdpi.com/2079-4991/10/11/2301/s1, Figure S1: Cytokine antibody array. Figure S2: IL-6 and CXCL2.
