# Supplementary material for: Biomimetic Nanoparticles Potentiate the Anti-Inflammatory Properties of Dexamethasone and Reduce the Cytokine Storm Syndrome: An Additional Weapon against COVID-19?
Source: Nanomaterials (Basel). 2020 Nov 20;10(11):2301. doi: 10.3390/nano10112301 (PMC7699958; doi:10.3390/nano10112301)
Supplement: Supplementary file 1 [file nanomaterials-10-02301-s001.pdf]

nanomaterials

Supporting Information

Biomimetic Nanoparticles Potentiate the Anti-Inflammatory Properties of Dexamethasone and Reduce the Cytokine Storm Syndrome: An Additional Weapon against COVID-19?

Roberto Molinaro [1,2], Anna Pasto [1], Francesca Taraballi [3,4], Federica Giordano [3,4], Jamil A. Azzi [5], Ennio Tasciotti [1,4,6] and Claudia Corbo [1,7*]

1 Center for Biomimetic Medicine, Houston Methodist Research Institute, Houston, TX 77030, USA; molinaro.roberto@hsr.it (R.M.); anna.pasto@humanitasresearch.it (A.P.); tasciottiennio@gmail.com (E.T.)
2 IRCCS San Raffaele Hospital, Milan, 20132, Italy
3 Center for Musculoskeletal Regeneration, Houston Methodist Academic Institute, Houston, TX 77030, USA; ftaraballi2@houstonmethodist.org (F.T.); fgiordano@houstonmethodist.org (F.G.)
4 Orthopedics and Sport Medicine, Houston Methodist Hospital, Houston, TX 77030, USA
5 Renal Division, Brigham and Women's Hospital, Harvard Medical School, Boston, MA 02115, USA; jazzi@bwh.harvard.edu
6 Biotechnology Program, San Raffaele University and IRCCS San Raffaele Pisana, Roma RM, 00166, Italy
7 Department of Medicine and Surgery, Center for Nanomedicine Nanomib, University of Milan Bicocca, Vedano al Lambro MB, 20854, Italy
* Correspondence: claudia.corbo@unimib.it

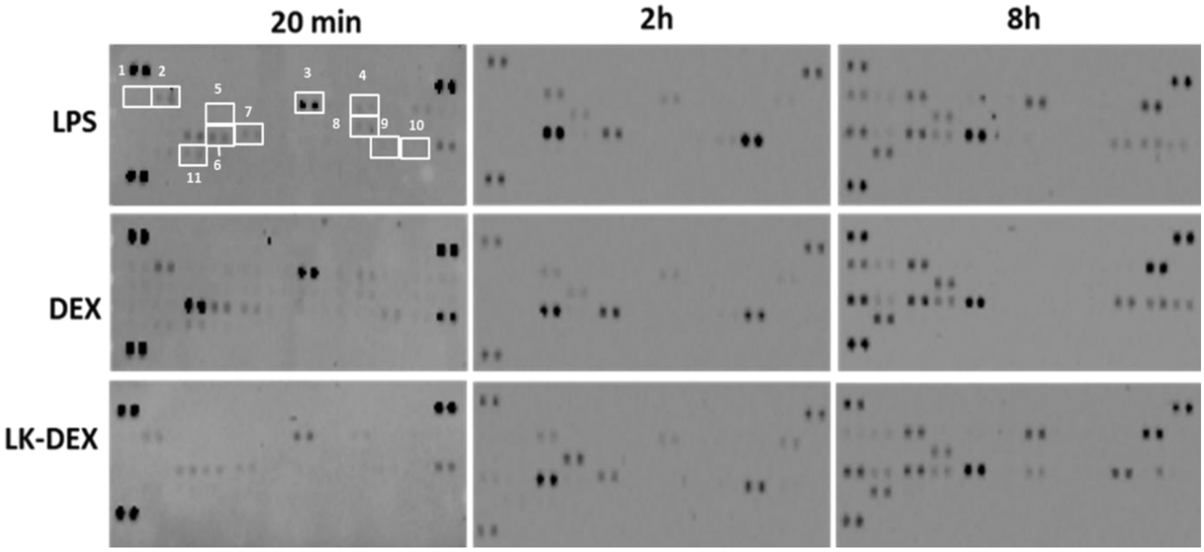

Figure S1. Cytokine antibody array**.** BALB/C mice were subjected to uncontrolled inflammation by injection of LPS and administrated with free dexamethasone, dexamethasone-loaded leukosomes or left untreated. Pooled plasma was used to evaluate the levels of cytokines by antibody array at 20 min, 2h and 8h post treatment. The cytokines are represented by duplicate spots in the locations shown: 1. BLC/CXCL13/BCA-1; 2. C5/C5a; 3. ICAM-1; 4. Il-1α; 5. IL-6; 6. M-CSF; 7. CCL2/MCP-1; 8. IL-16; 9. MIP-2/CXCL2; 10. RANTES; 11. TNF-α. Individual cytokines were identified following manufacturer's instructions.

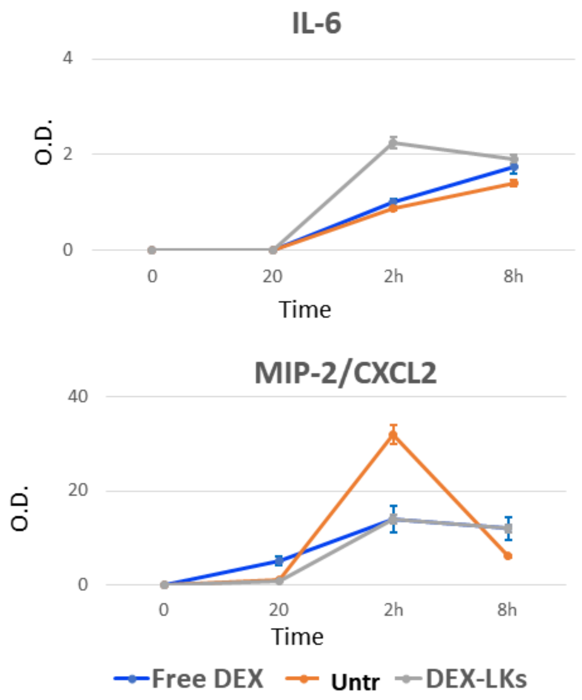

Figure S2. IL-6 and CXCL2. The average net optical intensities (O.D.) for IL-6 and CXCL2 is shown for each group at different time points. The basal levels of plasma cytokines of non-treated mice were evaluated by the same array and used to normalize the data. Results are presented as mean ± SD (n = 4).
